# Fatal and Nonfatal Drowning Outcomes Related to Dangerous Underwater Breath-Holding Behaviors — New York State, 1988–2011

**Published:** 2015-05-22

**Authors:** Christopher Boyd, Amanda Levy, Trevor McProud, Lilly Huang, Eli Raneses, Carolyn Olson

**Affiliations:** 1 Division of Environmental Health, New York City Department of Health and Mental Hygiene

Drowning is an important cause of preventable injury and mortality, ranking fifth among leading causes of unintentional injury death in the United States (1). In 2011, two healthy young men died in a drowning incident at a New York City (NYC)–regulated swimming facility. The men became unconscious underwater after performing intentional hyperventilation before submersion. The phenomenon of healthy swimmers becoming unconscious underwater has been described elsewhere as hypoxic blackout (2). Prompted by this incident, the NYC Department of Health and Mental Hygiene (DOHMH) in collaboration with the New York State Department of Health (SDOH) conducted a case review of New York state fatal and nonfatal drownings reported during 1988–2011 to investigate similar behaviors in other incidents. DOHMH identified 16 cases, three in NYC, with a consistent set of voluntary behaviors associated with unintentional drowning and designated this class of behaviors as “dangerous underwater breath-holding behaviors” (DUBBs). For this small sample, the frequency of different DUBBs varied by age and swimming level, and practicing more than one DUBB increased the risk for fatality. This research contributes to the literature on drowning by focusing on contributing behaviors rather than drowning outcomes. NYC recently enacted public health education and regulations that discourage DUBBs; these interventions have the potential to effectively reduce unintentional drowning related to these behaviors and could be considered by other municipalities and jurisdictions.

Drownings associated with DUBBs can occur at any water depth and be caused by many disparate factors. A precise definition describes the condition as “breath-hold blackout,” referring to the behavior rather than the outcome (i.e., hypoxic blackout) (3,4). The physiologic mechanism by which breath-hold blackout drownings occur is well-understood (5). Hyperventilation or breath-holding before diving or swimming decreases the body’s stores of CO_2_ and partial pressure of carbon dioxide (P_CO2_), delaying the cerebral response to come to the surface to breathe. The “blackout” is caused by the drop in partial pressure of oxygen (P_O2_) in arterial blood gas, resulting in hypoxia and loss of consciousness underwater. However, the behavioral antecedents of these drownings often go unreported. Only two case series studies from the 1960s and 1970s examined common features in drowning incidents in which hyperventilation resulted in the loss of consciousness underwater (5,6).

To identify incidents for this case series, the following list of three DUBBs was generated through a review of the available literature, expert opinion, and behaviors documented in identified cases (6,7): 1) intentional hyperventilation before or during submergence/swimming; 2) static apnea (i.e., when a swimmer submerges and attempts breath-holding for as long as possible, including “breath-holding games” with a partner, often while staying motionless); and 3) hypoxic training (i.e., prolonged underwater distance swimming or extended breath-hold intervals, which might be supervised or unsupervised).

Case information was developed by DOHMH from a review of incidents occurring at regulated swimming facilities (i.e., bathing establishments used for reasons other than personal or medical) using an SDOH database and available documentation from incident reports, lifeguard reports, police reports, inspection reports, emergency medical services reports, and hospital and medical examiner reports. Search terms used to identify fatal and nonfatal unintentional drowning cases based on the swimmer’s behavior included “repeated breath-holding,” “breath-holding games/competition,” “prolonged/extended submersion,” “underwater distance swimming,” and “hyperventilation.” DOHMH developed a case definition for DUBB-related incidents as those in which 1) fatal or nonfatal drowning followed one or more of the three DUBBs, and 2) the swimmer was otherwise not impaired and had no known preexisting health condition.

Six of 22 identified cases were excluded because of existing medical conditions or substance use, resulting in a case series of 16 DUBB-related drownings. Swimming ability was characterized as beginner, good, advanced, or unknown based on SDOH drowning investigation guidelines.

## Behavior Types

The following cases illustrate each DUBB as a contributing cause of unintentional drowning injury.

### Intentional hyperventilation

Two advanced-level, adult, male swimmers in good health were performing strenuous exercises to prepare for an advanced military fitness test. After alternating between push-ups and swimming laps, the swimmers began intentional hyperventilation and submersion breath-control exercises. Minutes later, both swimmers were found submerged underwater and not moving. Cardiopulmonary resuscitation was administered by lifeguards until emergency medical services personnel arrived. Both men were pronounced dead at the hospital.

### Static apnea

A teenage, male swimmer in good health with unknown swimming experience was participating in breath-holding contests and horseplay with friends. The swimmer fell unconscious underwater and his friends alerted lifeguards. Lifeguards were able to resuscitate him.

### Hypoxic training

An advanced-level, teenage, male swimmer with no preexisting health conditions and experience working as a lifeguard was training for his goal to join the U.S. Navy Seals. He was observed by pool staff performing breath-holding exercises and underwater lap swimming. He repeatedly submerged himself for extended periods of time, until it was noticed that he was unconscious. Efforts were made by the lifeguards and emergency medical services personnel to resuscitate him. He was pronounced dead at the hospital.

## Summary of 16 cases

The 16 DUBB cases in New York State during 1988–2011 involved 16 persons, 15 incidents (one of which included two persons), and four fatalities. Swimmers were aged 7–47 years, with an average age of 17 years (Table). Similar to most recorded drowning cases nationwide, the majority of the persons involved were male (n = 13). The most commonly reported DUBB behavior was static apnea (n = six). Four cases were associated with hypoxic training, three cases with intentional hyperventilation, and two fatal cases had a combination of both intentional hyperventilation and hypoxic training behaviors. In all four fatalities, the decedents were aged 17–22 years, known to be advanced to expert swimmers, and engaged in intentional hyperventilation. As illustrated in the static apnea incident described previously, half of decedents engaged in a DUBB coactivity (multiple behaviors) with intentional hyperventilation and underwater lap swimming.

DUBBs differed by both swimming experience and age group. Swimming experience was known for 14 cases. All intentional hyperventilation cases and half of all DUBB incidents involved advanced-level swimmers. Among swimmers with known experience (all aged 7–24 years), more experienced swimmers (n = eight) engaged in hypoxic training, intentional hyperventilation or both, whereas less experienced swimmers engaged primarily in static apnea.

Of the 16 drownings, 15 occurred at a pool facility: seven outdoors, seven indoors, and one in a wave pool. The remaining incident occurred in a nonregulated stream. More than half of all DUBB incidents occurred when more than one swimmer was in the pool with the affected swimmer at the time of the incident.

What is already known on this topic?

Drowning is a major source of injury in the United States. The physiologic causes of drownings related to breath-holding among otherwise healthy swimmers have been the focus of aquatic program–based materials on drowning prevention and academic literature, but little research has examined the epidemiology of contributing behaviors in such incidents.

What is added by this report?

This report identifies a class of swimming behaviors, designated dangerous underwater breath-holding behaviors (DUBBs), that can lead to potentially fatal drowning outcomes and could be easily prevented to decrease the risk for drowning among otherwise healthy swimmers. They include intentional hyperventilation, static apnea, and hypoxic training. The frequency of different DUBBs varied by age and swimming level of the swimmers involved, and for this small sample, practicing more than one DUBB type increased the likelihood of a drowning injury.

What are the implications for public health practice?

Drowning continues to present a public health risk, even in facilities that have adequate lifeguards and other safety precautions. Through educational initiatives and policy-level changes to the New York City Health Code, the New York City Department of Health and Mental Hygiene has taken steps to increase awareness of dangerous swimming behaviors to prevent unintentional drownings.

All but one of the incidents at the 15 regulated facilities occurred with a lifeguard on duty and involved a lifeguard rescue attempt. The exception was an incident during which a member of an advanced high school swimming program was practicing hypoxic behavior at his school’s private facility before hours of operation.

### Discussion

This case series highlights a group of voluntary, dangerous behaviors that contributed to a number of unintentional drownings in New York State. The findings contribute to drowning prevention research by shifting focus from final outcomes to modifiable behavioral risk factors. DUBBs can lead to drowning in otherwise healthy persons, so incidence of this type of drowning can be prevented with interventions such as improved supervision, regulation, and public education (8). Since 2014, the NYC Health Code requires the posting of prevention-focused signage at permitted bathing establishments, with warnings that intentional hyperventilation and competitive, repetitive, or prolonged underwater swimming or breath-holding can be dangerous. The code also requires that facilities post a pictorial warning sign aimed at younger swimmers, and it expands pool operator responsibilities to include discouraging such DUBBs and updating their site safety plans to prohibit DUBBs unless explicitly permitted under enhanced supervision. Future intervention activities will include educational efforts to inform parents, coaches, safety officials, and swimmers about the risks for DUBBs.

The findings in this report are subject to at least two limitations. First, because this study used incident reports as surveillance data, changes in definitions and coding conventions during the 20-year timeframe might have led to some missed cases. Second, cases might have been missed because behaviors leading to drownings are frequently underreported. Fifteen of the 16 incidents in this case study occurred at bathing facilities that require an operating permit from DOHMH, and all had witnesses who reported predrowning behaviors. However, research suggests that more than half of drowning incidents are not witnessed (9,10). A previous case study found that swimmers who engage in the most dangerous DUBB (intentional hyperventilation) might do so regularly (9), suggesting the possibility of unobserved incidents.

## Figures and Tables

**TABLE:** Summary of dangerous underwater breath-holding behaviors (DUBBs) resulting in fatal or nonfatal drowning, by selected characteristics — New York State, 1988–2011

Characteristic	Total		DUBB type							

			Intentional hyperventilation		Static apnea		Hypoxic training		Intentional hyperventilation and hypoxic training coactivity	
				
	No.	(%)	No.	(%)	No.	(%)	No.	(%)	No.	(%)
**Overall**	**16**	**(100)**	3	(19)	6	(38)	5	(31)	2	(13)
**Sex**										
Male	**13**	**(81)**	3	(100)	4	(67)	3	(60)	2	(100)
Female	**3**	**(19)**	0	—	2	(33)	1	(20)	0	—
**Age group (yrs)**										
<15	**7**	**(44)**	0	—	4	(67)	3	(60)	0	—
15–24	**8**	**(50)**	2	(67)	2	(33)	1	(20)	2	(100)
≥25	**1**	**(6)**	1	(33)	0	—	0	—	0	—
**Drowning type**										
Nonfatal drowning	**12**	**(75)**	1	(33)	6	(100)	5	(100)	0	—
Fatal drowning	**4**	**(25)**	2	(67)	0	—	0	—	2	(100)
**Swimming ability**										
Beginner	**5**	**(31)**	0	—	5	(83)	0	—	0	—
Good	**1**	**(6)**	0	—	0	—	1	(20)	0	—
Advanced	**8**	**(50)**	3	(100)	0	—	3	(60)	2	(100)
Unknown	**2**	**(13)**	0	—	1	(17)	1	(20)	0	—
**Facility type**										
Public	**7**	**(47)**	3	(100)	0	—	2	(40)	2	(100)
Private	**9**	**(53)**	0	—	6	(100)	3	(60)	0	—
**Bathing facility type**										
Indoor pool	**7**	**(44)**	1	(33)	2	(33)	3	(60)	1	(50)
Outdoor pool	**7**	**(44)**	2	(67)	2	(33)	2	(40)	1	(50)
Wave pool	**1**	**(6)**	0	—	1	(17)	0	—	0	—
Nonregulated stream	**1**	**(6)**	0	—	1	(17)	0	—	0	—
**No. of other bathers at time of incident**										
None	**1**	**(6)**	1	(33)	0	—	0	—	0	—
1–5	**2**	**(13)**	0	—	1	(17)	0	—	1	(50)
6–10	**0**	**—**	0	—	0	—	0	—	0	—
11–20	**6**	**(38)**	2	(67)	2	(33)	1	(20)	1	(50)
>20	**1**	**(6)**	0	—	0	—	1	(20)	0	—
Unknown	**6**	**(38)**	0	—	3	(50)	3	(60)	0	—
**No. of lifeguards on duty at time of incident**										
None	**1**	**(6)**	0	—	0	—	1	(20)	0	—
1–2	**8**	**(50)**	0	—	2	(33)	2	(40)	1	(50)
3–5	**2**	**(13)**	0	—	1	(17)	0	—	1	(50)
>5	**0**	**—**	0	—	0	—	0	—	0	—
Unknown	**5**	**(31)**	3	(100)	3	(50)	2	(40)	0	—
